# Cognitive mechanisms of statistical learning and segmentation of continuous sensory input

**DOI:** 10.3758/s13421-021-01264-0

**Published:** 2021-12-29

**Authors:** Leona Polyanskaya

**Affiliations:** Departamento de Psicología y Sociología, Universidad de Zaragoza, Teruel, Spain

**Keywords:** Statistical learning, Word segmentation, Sequence learning, Clustering, Boundary-finding, Artificial language

## Abstract

Two classes of cognitive mechanisms have been proposed to explain segmentation of continuous sensory input into discrete recurrent constituents: clustering and boundary-finding mechanisms. Clustering mechanisms are based on identifying frequently co-occurring elements and merging them together as parts that form a single constituent. Bracketing (or boundary-finding) mechanisms work by identifying rarely co-occurring elements that correspond to the boundaries between discrete constituents. In a series of behavioral experiments, I tested which mechanisms are at play in the visual modality both during segmentation of a continuous syllabic sequence into discrete word-like constituents and during recognition of segmented constituents. Additionally, I explored conscious awareness of the products of statistical learning—whole constituents versus merged clusters of smaller subunits. My results suggest that both online segmentation and offline recognition of extracted constituents rely on detecting frequently co-occurring elements, a process likely based on associative memory. However, people are more aware of having learnt whole tokens than of recurrent composite clusters.

Although sensory input is continuous, cognitive systems operate on discrete constituents. Splitting continuous sensory input into discrete units is called *segmentation*. Segmentation is based on statistical learning, the process of detecting statistical regularities in continuous sensory input in order to structure this input into processable units. Statistical learning mechanisms operate across all modalities (visual, auditory, tactile: Conway & Christiansen, 2006; Kirkham et al., 2002); and across domains (splitting speech into words and phrases: Thiessen et al., 2013: music compositions into rhythmic groups: Deutsch, 2013; Povel & Essens, 1985; Ravignani et al., 2016; continuous actions into discrete event sequences: Hard et al., 2019, among others).

Statistical learning mechanisms are very important for language learning and speech processing (Erickson & Thiessen, 2015; Misyak & Christiansen, 2012). They are engaged in multiple language-related tasks: category formation and detection (Erickson et al., 2014; Maye et al., 2002); encoding and decoding of linguistic sequences (Stadler, 1992); rule learning and cause–consequence understanding (Sobel & Kirkham, 2007); and associations between real-world objects and discrete constituents segmented from continuous acoustic streams (Graf Estes et al., 2007; Smith & Yu, 2008). Therefore, better knowledge of the cognitive mechanisms underlying statistical learning, including segmentation, is crucial to understanding how humans process language. In this study, I will focus on a subtype of statistical learning based on conditional statistics—that is, the predictive relationships between two syllables (Harris, 1955). The strength of predictive relationships is measured as the transitional probability (TP) between adjacent syllables (Saffran et al., 1996). TPs tend to be higher between syllables within word boundaries than between syllables straddling word boundaries, and this difference can be used to extract words as discrete constituents from a continuous stream of syllables. Note that statistical structures include both conditional and distributional information (e.g., prosody, pauses, coarticulatory differences in phoneme realization both at word boundaries and within words) and can be considered within a single theoretical framework. Although distributional cues are extensively used for segmenting natural speech (Mattys et al., 1999), this study focuses exclusively on cognitive mechanisms for processing conditional statistics.

The cognitive mechanisms used to extract, learn, and employ conditional statistics are highly debated. Two possible classes of mechanisms, which assign different roles to TPs in the segmentation of continuous input, have been proposed: (1) clustering mechanisms and (2) boundary-finding mechanisms (see Perruchet & Pacton, 2006, and Perruchet, 2019, for an overview). Boundary-finding mechanisms (often referred to as *bracketing* mechanisms) are based on detecting high-entropy transitions, where one element predicts the following element with low probability. This is a general mechanism for detecting rarely co-occurring elements, and high-entropy transitions between elements signal boundaries between discrete constituents (Elman, 1990). By contrast, clustering mechanisms work by detecting element pairs with high transitional probabilities (i.e., detecting those clusters of elements that frequently co-occur together; Frank et al., 2010; Perruchet & Vinter, 1998).

While high TPs correspond to syllable pairs within the boundaries of discrete constituents (e.g., words or phrases), extraction need not rely on the calculation of these TPs, because frequently co-occurring elements can be committed to memory based on associative learning mechanisms (Perruchet, 2019; Perruchet & Vinter, 1998). In fact, statistical learning abilities could, more generally, be based on a set of memory mechanisms rather than extraction of statistical structures and regularities (McClelland et al., 1995; Thiessen, 2017).

In a critical review, Perruchet (2019) analyzed recent evidence and concluded that transitional probabilities are neither sufficient nor necessary for segmentation, since memory mechanisms can account for both conditional and distributional statistical learning (Thiessen, 2017). This proposal has received support from computational models of segmentation (Christiansen et al., 1998; Perruchet & Vinter, 1998). However, some evidence suggests that low TPs attract the attention of infants who are just beginning to acquire language and need to extract lexical units from continuous speech before they can figure out the semantic content and reference of these units (Endress & Langus, 2017; Endress & Mehler, 2009a, 2009b; Johnson et al., 2009). Thus, it is possible that both types of mechanisms operate in segmentation (Sohail & Johnson, 2016, for edge detection, and Slone & Johnson, 2018, for clustering by babies), depending on the environment and the nature of the constituents that need to be segmented from the sensory input. Modality may also play a role in determining which mechanisms are more likely to be engaged. In the visual modality, for example, when several elements are presented simultaneously, recurrent patterns might more easily be registered as frequently co-occurring configurations (Fiser & Aslin, 2005; Glicksohn & Cohen, 2011); in the auditory modality, consecutively presented sequences of syllables might more easily engage boundary-finding mechanisms (e.g., Ordin, Polyanskaya, Soto, & Molinaro, 2020b). Giroux and Rey (2009) showed that after 2 minutes of exposure to an artificial language, participants were equally likely to prefer bisyllabic and trisyllabic words, as well as pairs from which trisyllabic words were composed, over part-words (syllabic concatenations composed of a final syllable from one recurrent word and the initial syllable of a different word from the familiarization input). Only after 10 minutes of exposure was the discrimination of part-words and words better than discrimination of part-words and subword syllable pairs. This clearly shows that at least at the initial stages of segmentation, recurrent syllable pairs were the perceptual units that were extracted and memorized. Longer exposure was necessary for longer sequences, composed of several recurrent pairs, to be learnt. When complex words had been memorized, representation of their subcomponents (the syllable pairs from which trisyllabic words were composed) was impeded. A fundamental difference between clustering mechanisms operating in the visual and auditory modality is that in the visual modality, complex scenes can be learnt without acquiring smaller subconstituents (Fiser & Aslin, 2005; Glicksohn & Cohen, 2011). By contrast, in the auditory modality, subconstituents are extracted and memorized quickly, before representations of longer recurrent sequences can be formed (Giroux & Rey, 2009). It is possible that complex scenes composed of simultaneously presented elements are segmented exclusively by clustering mechanisms, while sequences from consecutively presented elements are learnt using clustering mechanisms at the beginning but later, when constituents have been memorized and their representations are well-established, they can be segmented from continuous input based on boundary-finding mechanisms.

The current study was designed to address two issues: What cognitive mechanisms are brought to bear during the segmentation of linguistic input in the visual modality? And to what extent are people consciously aware of the constituents that emerge as psychological units over the course of visual familiarization with a continuous stream of syllables? In this study, a standard artificial language learning paradigm (Saffran et al., 1996) was used. For the familiarization stream, 2 trisyllabic words were concatenated, with 0.5 TPs between syllables within word boundaries, and visually presented this stream to participants. Participants were told that the stream represented a text in an unfamiliar language. For the subsequent recognition test, the same syllables were used to construct novel items, which were not embedded in the familiarization stream, but embodied the same TPs as the words. For example, the syllables from the words RO-SE-NU and PA-SE-TI (0.5 TPs between adjacent syllables within triplets) were recombined to create novel tokens—RO-SE-TI and PA-SE-NU. These novel tokens were statistically congruent with the words yet could not be retrieved from memory as whole constituents because they had not been embedded in the familiarization stream as whole triplets. However, participants might still endorse these novel items (*phantoms*) because they were statistically consistent with the exemplars in the familiarization input (e.g., Endress & Langus, 2017; Endress & Mehler, 2009a; Nosofsky & Zaki, 2002; Roediger & McDermott, 1995), or because they were composed of frequently co-occurring syllable pairs (Perruchet, 2019; Thiessen, 2017). For the recognition test, three types of tokens were used: old syllabic triplets from the familiarization stream (*words*), *phantoms*, and novel triplets that violated the statistical regularities of the familiarization input (*nonwords*). During the test, participants performed a binary yes/no test (deciding whether or not the presented syllabic triplet was a word from the language they had been familiarized with). In Experiment 1, the test tokens were presented in isolation (as a three-syllabic sequence). Here, participants could only rely on the TPs (0.5 for words or 0 for nonwords) between syllables within presented tokens, which were either 0.5 or 0 between the first and second and the second and third syllables. Since participants had to rely on mid-token TPs, they had to base their decision on its absolute value (high vs. low). By contrast, in Experiment 2, participants saw seven syllables on the screen and had to report whether a recurrent triplet from the familiarization stream was embedded within a seven-syllable sequence. Here, participants could also rely on the relative differences between TPs, since TPs between syllables straddling the triplet boundary were lower than TPs between syllables within words and phantoms. Relatively lower TPs marked triplet boundaries and therefore provided better conditions for engaging boundary-finding mechanisms. Additionally, in both experiments, conscious awareness of the decisions made during the test was probed. This should cast light on the contribution of consciousness to recognition of discrete tokens retrieved from memory as whole constituents (i.e., *words* that were embedded as recurrent triplets in the familiarization input) or were reconstructed during the recognition test based on clusters of frequently co-occurring syllables (without additional support from memory representations of whole triplets).

To understand the cognitive mechanisms that underpin segmentation and recognition of segmented constituents, these behavioral measures and eye-tracking measures were combined. This is the first study to use eye-tracking to resolve the respective contributions of clustering and boundary-finding mechanisms to statistical learning. The saccade behavior was explored as a proxy for attention to reveal underlying cognitive mechanisms. It should reveal whether participants attended—that is, made more frequent saccades between co-occurring syllables or instead to the boundaries (edges) of the recurrent embedded constituents. The expectations were as follows. In Experiment 1, clustering mechanisms would elicit saccades between adjacent syllables, while bracketing mechanisms would elicit saccades between the initial and final syllables of the triplets. In Experiment 2, bracketing mechanisms would elicit saccades to syllable boundaries with lower TPs (i.e., short range, pairwise saccades to and from the triplet-initial and preceding syllables or to and from the triplet-final and following syllables), and longer saccades across a triplet (i.e., between the triplet-initial and triplet-final syllables) to check triplet boundaries (edges). Clustering mechanisms would instead elicit saccades to and from syllables within triplets to check that syllables within each pair were indeed frequently co-occurring. Saccades were analyzed separately for both words and phantoms, since endorsement of different token types might be based on different mechanisms: words might be accepted based on boundary detection, while phantoms might be endorsed based on clustering; if so, differences in saccade behavior on different types of statistically congruent tokens might be observed. Eye-tracking provides exquisite temporal and spatial resolution. The analysis of saccades allows for precise identification of the time and location of saccade start points and end points. Thus, this technique can be useful for identifying which cognitive mechanisms are deployed under various conditions.

For the other behavioral measures, the conditional measures of accuracy and the confidence judgements assigned to each response were used. Confidence that discriminates between correct and wrong responses reflects awareness of one’s decisions (Fleming & Lau, 2014; Schwiedrzik et al., 2011). Even if recognition responses are based on information that is not subject to conscious processing, participants have to rely on information that is consciously processed when they report confidence judgments (how sure they are that their response is correct; Del Cul et al., 2009; Pasquali et al., 2010). Thus, confidence ratings can reveal the relative contribution of conscious content to correct decisions on words, phantoms, and nonwords. Perruchet and Pacton (2006) proposed two alternatives: Chunks might be formed as a result of unconscious processing, or conscious chunks may emerge at the beginning, but later be merged into larger units as a result of associative learning. If people are aware of chunks that comprise larger constituents, a large difference between confidence ratings for accepted and rejected phantoms—similar to the difference between accepted and rejected words—should be observed. Note that the smallest difference in confidence ratings was expected between accepted and rejected nonwords, because in the case of nonwords, people have to report their confidence about something they have never learnt (they have not learnt nonwords, and hence cannot be consciously aware of nonwords as perceptual units).

## Experiment 1

### Methods (Experiment 1)

Experiment 1 focused on the *recognition* of holistic constituents from the familiarization stream, presented in isolation. Both experiments were approved by the ethical review panel of the Basque Centre on Cognition, Brain and Language (Ethics Approval Number: 17092018M).

#### Participants

The sample size was determined based on earlier studies conducted in a different (auditory) modality with different techniques (EEG and fMRI) but the same materials (*N* = 34 in Ordin & Polyanskaya, 2021, and Ordin, Polyanskaya, Soto, & Molinaro, 2020b; *N* = 25 in Ordin, Polyanskaya, & Soto, 2020a). Despite important differences in modality and techniques, these studies provided important indications for the sample size necessary to obtain significant results—if there were any—in behavioral measures, including recognition accuracy and confidence ratings. Forty participants with normal or corrected-to-normal vision were recruited for this study. All participants were students at the University of the Basque Country (age range: 18–35 years, *M* = 24, 26 females). All had lived in a bilingual (Basque–Spanish) environment from birth and were fluent and regular users of both languages across all communication contexts. Relative proficiency in both languages was assessed by lexical access. Participants had to name 65 objects first in Basque, then in Spanish; all object names were noncognates, and participants received one point for each correctly named object. The difference between the scores achieved in Basque and Spanish was used as a proxy for any proficiency skew towards one of these languages. However, all participants achieved ceiling scores in both languages, reflecting high proficiency across both of their two languages. All participants had at least school-based knowledge of English. Importantly, the core learning ability to extract and employ conditional and distributional statistical cues for segmentation of continuous sensory input is known to be similar across speakers of different languages and is not affected by bilingualism (Ordin et al., 2017; Poepsel & Weiss, 2016; Yim & Rudoy, 2013); thus, the reported results are expected to generalize over native speakers of other languages and also over monolingual populations.

#### Material

**Fig. 1:**
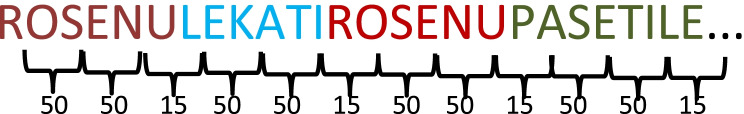
TPs between adjacent syllables: TPs between syllables within triplets are higher; TPs between syllables straddling triplet boundaries are lower

A syllable inventory of 18 consonant–vowel (CV) syllables was used to construct 12 trisyllabic statistical nonsense words with a unique combination of consonants, each syllable contributing to two statistical *words*. The positions of plosive, sonorant, and fricative consonants were counterbalanced across words; each consonant was used an equal number of times. Twelve statistical words were randomly concatenated 68 times, with each word repeated only once per block. These 68 blocks of 12 words were then randomly concatenated, ensuring that no word was ever repeated twice consecutively (at the block boundaries). In the resulting stream of syllables, which was used as a familiarization stream, TPs between syllables within words were 50%, and TPs between syllables straddling statistical word boundaries were around 15%. Higher TPs (between syllables within triplets) could be used to merge syllables as parts of the same constituent, while lower TPs would signal boundaries between recurrent triplets (see Fig. 1). This network of TPs allowed for the emergence of perceptual units that will be referred to as *phantoms* (three-syllabic sequences that did not appear in the habituation sequence of syllables as whole triplets, yet had 50% TPs between syllables, and were composed of the same syllable pairs that constituted the words). For the recognition test, 12 *nonwords* were created by concatenating the syllables randomly, so that none of the syllable pairs in nonwords occurred consecutively in the familiarization stream.

The list of words, phantoms, and nonwords is given in Table 1.

**Table 1:** List of words, phantoms, and nonwords

Words	Phantoms	Nonwords
ROSENU ROKAFA PASETI LEKATI PAMONU LEMOFA PERIKO MURIFO PETASA LUTAFO MUNIKO LUNISA	PASENU LEKAFA ROSETI ROKATI PAMOFA LEMONU MURIKO LUTASA PERIFO PETAFO MUNISA LUNIKO	ROTIMO SEPAKO FALUSA FOLERI TAMUPE NIKANU NURIPE FOLUKA NIMUKO MOPERO LESATI TASEFA

#### Procedure

The experiment consisted of two parts: a learning session, immediately followed by a recognition test. Participants sat in front of the computer 60 cm from a 19-in. CRT Viewsonic monitor with their head stabilized by a chin rest and forehead restraint bar. Eye-movements were tracked using an SR EyeLink 1000 machine. The experimental room was dimmed to provide better viewing and more efficient eye-tracking. Calibration and validation were carried out using a standard 9-point procedure (9-point grid calibration, biquadratic with corner correction). The stimuli were presented via Experiment Builder Software (SR Research, Ontario, Canada). Participants were informed that they were going to see a text in an unfamiliar language, presented without pauses between the words. They were told that they should try to detect and memorize the words of this unfamiliar language.

**Fig. 2:**
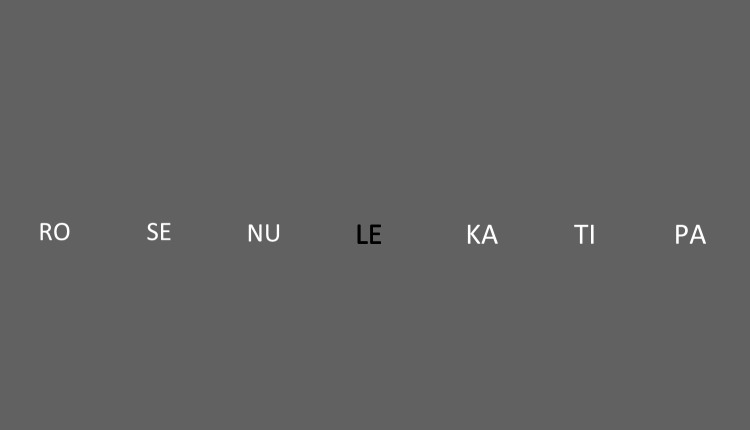
Schematic illustration of the screen view presented to the participants during the learning stage. The colors of the fonts and the background are similar to those used in the actual experiment. The syllables were displayed at 4-cm intervals along the central horizontal line. Each screen was presented for 500 ms, then the syllables were moved leftwards, so that the leftmost syllable disappeared and the subsequent syllable appeared on the right (in the example, the syllable “MO,” the next element in the PAMONU triplet, would appear on the right)

The screen was divided into seven interest areas (Fig. 2), and each region was populated with a syllable. The syllables moved to the left every 500 ms, and the rightmost area of interest then displayed the following syllable in the learning sequence. The first and the last few syllables in the sequence were dimmed and gradually became obvious (at the beginning) or more obscure (at the end), preventing the participants from identifying the initial and final syllables as boundary markers for future segmentation. The syllables were presented in the Arial font (letter height: 2 cm, 4 cm between each syllable), in black (central position) or white (peripheral positions) against a grey background.

Following the learning session, participants performed a recognition test, during which eye-tracking data were collected. The right eye was tracked for all participants. Participants had to look at the trisyllabic sequence—syllables were distributed linearly, with 8 cm between the consecutive syllables—and respond whether the triplet was an actual word from the language they had learnt. They then had to assign a confidence rating—on a 6-point scale—to indicate how sure they were about their response. The syllables were presented for 2,000 ms, and the time for response was not limited. The responses were given using a mouse. The presented sequences could be either *words* (triplets that recurrently occurred during familiarization), or *phantoms* (triplets that never occurred during familiarization as whole constituents, but conformed to the same statistical regularities as words), or *nonwords* (novel triplets composed of syllable pairs that had never occurred consecutively during familiarization (i.e., in which TPs between adjacent syllables were zero). The position of yes/no buttons was counterbalanced, while the direction of the confidence scale was maintained for all participants because it is more natural to assign a lower value (e.g., “1”) to a lower confidence and a higher value (e.g., “6”) to a higher degree of confidence, and people are more used to tracking ascending numbers (representing the ascending degrees of confidence) from left to right. In sum, it was considered necessary to counterbalance yes/no positions, while counterbalancing the order of responses on the confidence scale was deliberately avoided.

**Fig. 3:**
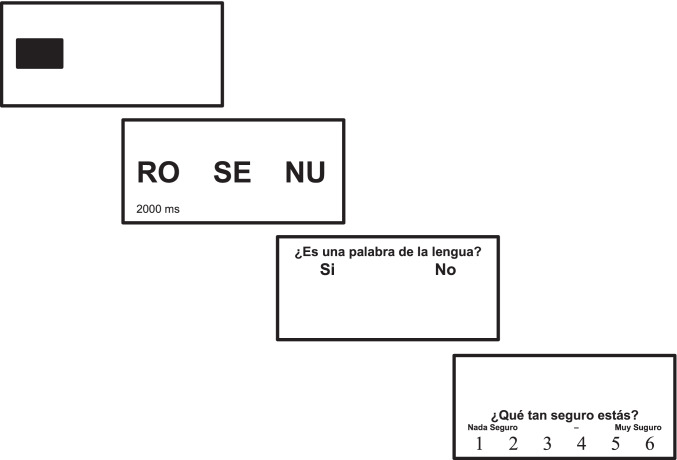
The schematic representation of the experimental trial during the recognition test, Experiment 1. The questions—in Spanish—were (1) Is it a word from the language? ( “yes” or “no”), and (2) How sure are you? (1 = *not sure at all* to 6 = *very sure*)

For the test, 36 test tokens (12 words, 12 phantoms, 12 nonwords) were randomized and sequentially presented to the participants three times (108 test trials in total). The eye-tracking machine was calibrated before the recognition test, and the calibration procedure was repeated after each block of 36 trials, using the same 9-point grid calibration-validation procedure. Each trial was initiated as soon as the participant fixated the black square marking the place where the first syllable of a triplet would appear. The structure of the trial and the experimental interface are presented in Fig. 3. Although it might have been difficult for participants to hold the familiarization words in memory over 108 trials (that were interrupted twice for recalibration), I did not observe any significant difference in performance between blocks for either type of test token (*word, phantoms, nonwords*). Afterwards, the numbers of endorsed and rejected words, phantoms and nonwords were averages across three blocks, to diminish any potential undetected effect of token repetitions.

### Results (Experiment 1)

#### Accuracy and confidence analysis

Forty participants were tested and two excluded (for pressing the same response button on all 108 trials, both for recognition and confidence questions). Further screening of confidence ratings and the number of “yes” and “no” responses in each category (words, phantoms, nonwords) revealed no outlying scores (defined as deviations exceeding two standard errors from the mean).

**Fig. 4:**
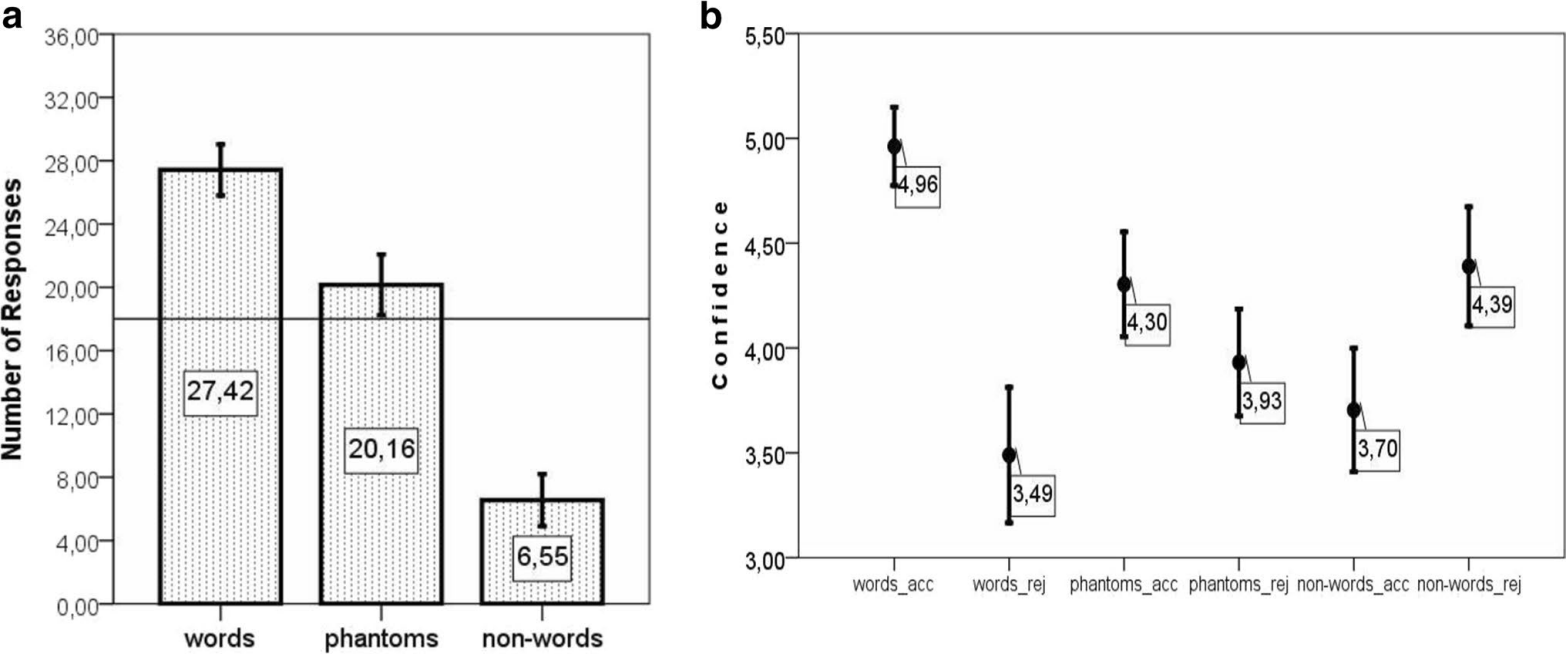
**a** Experiment 1: Accepted words, phantoms, and nonwords out of 36 trials per category of tokens. Error bars represent 95% CI. The horizontal line represents a chance-level response. **b** Experiment 1: Averaged confidence on accepted (acc) and rejected (rej) words, phantoms, and nonwords. Error bars represent 95% CI. The scale is cropped. The range for confidence ratings is from 1 (*minimum confidence*) to 6 (*maximum confidence*)

A series of one-sample *t* tests showed that the number of endorsed words, phantoms, and nonwords significantly differed from what would be expected by chance (50%, or 16 responses). Participants accepted words, *t*(37) = 14.38, *p* < .0005, *M* (mean difference from chance) = 11.42, 95%CI of the difference [9.81, 13.03], and phantoms, *t*(37) = 4.39, *p* < .0005, *M* = 4.16, [2.24, 6.08], significantly above chance and rejected nonwords, *t*(37) = −11.65, *p* < .0005, *M* = −9.45, [−11.09, −7.8]. As performance at the group level was different from what would be expected by chance, I suggest that these responses were not given randomly. The pattern of responses is represented in Fig. 4a.

A repeated-measures ANOVA on the number of endorsed words, phantoms, and nonwords showed a significant effect of category, *F*(2, 74) = 184.09, *p* < .0005, η_p_^2^ = .83 (λ = 368.18, noncentrality parameter). Pairwise tests (all pairwise tests are reported after Bonferroni correction) showed that differences between the number of accepted words and phantoms, *t*(37) = 6.96, *p* < .0005, *M* = 7.26, [5.15, 9.38], the number of accepted words and nonwords, *t*(37) = 16.13, *p* < .0005, *M* = 20.87, [18.25, 23.49], and the number of accepted phantoms and nonwords, *t*(37) = 14.39, *p* < .0005, *M* = 13.61, [11.69, 15.52], were significant. The difference between correct endorsements and correct rejections (i.e., between accepted words and rejected nonwords) was insignificant, *t*(37) = 2.131, *p* = .12.

For the confidence analysis (Fig. 4b), the difference in confidence assigned to accepted and rejected items in each category was compared. Two-tailed *t* tests showed that accepted words, *t*(37) = 9.87, *p* < .0005, *M* = 1.47, [1.17, 1.77], and accepted phantoms, *t*(37) = 2.88, *p* = .007, *M* = 7.26, [.11, .64], received higher confidence ratings than the corresponding rejected words and phantoms. Accepted nonwords were assigned lower confidence ratings, *t*(32) = 3.472, *p* = .002, *M* = .57, [.23, .9], than rejected nonwords. Please note that five participants did not endorse a single nonword, and this explains the lower degrees of freedom in the last test.

The differences in confidence ratings assigned to accepted and rejected tokens per category were subjected to a repeated-measures ANOVA with Greenhouse–Geiser correction (ε = .775) for sphericity violations. This analysis showed that the difference in confidence assigned to accepted and rejected tokens of different categories was significant, *F*(2, 64) = 12.41, *p* < .0005, η_p_^2^ = .279 (λ = 24.817) (corrected *p* values and uncorrected degrees of freedom are reported). Further pairwise comparisons (Bonferroni correction applied in all pairwise t-tests in the manuscript) showed that the difference in confidence assigned to endorsed and rejected words was significantly larger than that for accepted and rejected phantoms, *t*(37) = 6.85, *p* < .0005, *M* = 1.1, [.77, 1.42], or for accepted and rejected nonwords, *t*(32) = 4.04, *p* < .0005, *M* = .87, [.43, 1.31]. The difference in confidence assigned to accepted and rejected phantoms and accepted and rejected nonwords was not significant, *t*(32) = .542, *p* = .591, *M* = .15 [−.7, .4].

#### Saccade analysis

Prior to the fixation sequence analysis, the eye-tracking data were subject to a multi-stage fixation cleaning algorithm, which allows for refined data pre-processing and is recommended for analysis of eye movements in reading, implemented in the EyeLink software. At the first stage, fixations shorter than 80ms were merged with any neighboring fixation exceeding 80 ms as long as the distance between them did not exceed 0.5 degrees. At the second stage, the remaining shorter fixations (up to 40 ms) were merged with neighboring fixations at a distance of up to 1.25 degrees. At the next stage, fixations shorter than 100 ms and longer than 800 ms within a single interest area were deleted. The fixation sequence analysis was performed in the 2,000 ms time window, during which the syllabic triplet was presented.

**Fig. 5:**
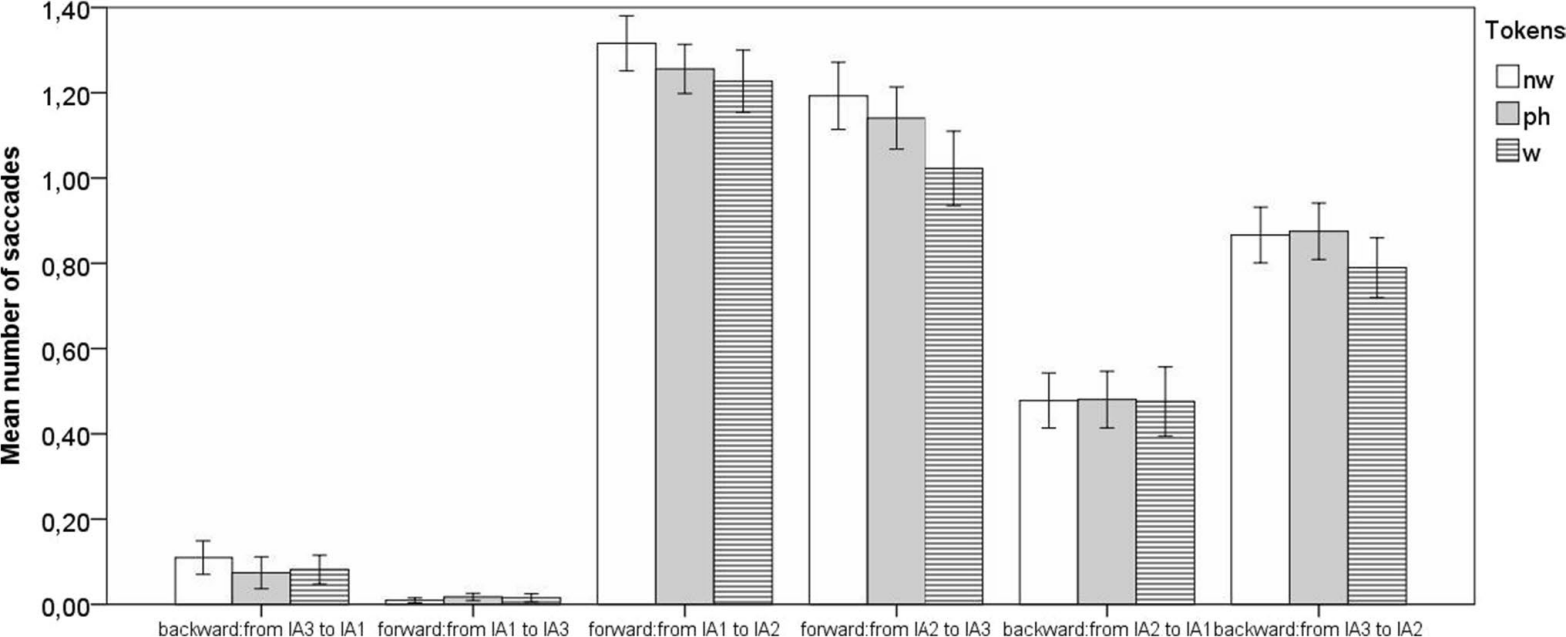
**a** Experiment 1: Mean number of saccades per trial between adjacent and boundary syllables in triplets split by token category. *Note.* nw = nonwords; ph = phantoms; w = words. Error bars represent 95% CI

After preprocessing, the saccades between interest areas (IAs) were split into pairwise forward saccades (from IA1 to IA2, from IA2 to IA3, and from IA1 to IA3), boundary-wise forward saccades (from IA1 to IA3), pairwise backward saccades (from IA3 to IA2 and from IA2 to IA1), and backward boundary-wise saccades (from IA3 to IA1). A single syllable fit one IA (see Methods section). Mean numbers of each saccade type for trials with words, phantoms, and nonwords were calculated and are plotted in Fig. 5a.

Forward pairwise saccades (from IA1 to IA2 and from IA2 to IA3) were the most frequent, while backward pairwise saccades, representing the cases when participants looked back at the preceding syllable, probably to verify their decisions, were less frequent. Of the latter, the backward saccades from the last to the middle syllable in the triplet were more frequent. Saccades between the boundary syllables were exceptionally rare. This pattern suggests that participants were making recognition decisions based on pairwise syllable combinations.

Further analysis was performed to identify whether saccades differed between words, phantoms, and nonwords. A repeated-measures ANOVA on each saccade type with *category* (words, phantoms, and nonwords) as a three-level factor showed that only the mean number of forward and backward saccades between IA2 and IA3 differed across categories (see Table 2), with phantoms eliciting more pairwise saccades than words. The numbers of endorsed phantoms and words differed, so that the fixation sequence could potentially be associated with the response. Thus, 2 × 2 repeated-measures ANOVAs, with *category* (words vs. phantoms) and response (accept vs. reject) as factors, were run. These tests showed that the effect of the response type (accept vs. reject) was significant neither for forward saccades from IA2 to IA3, *F*(1, 37) = 2.47, *p* = .25 (corrected), η_p_^2^ = .063, nor for backward saccades from IA3 to IA2, *F*(1, 37) = 3.42, *p* = .146 (corrected), η_p_^2^ = .085. Also, the interaction between response and token category (words vs. phantoms) was significant neither for backward (*p* = .958) nor for forward (*p* = .152) saccades. This suggests that the significant difference in the number of saccades between words and phantoms was not modulated by response type. The ANOVAs revealed that phantoms elicit more backward, *F*(1, 37) = 13.08, *p* = .002 (corrected), η_p_^2^=.261, and forward, *F*(1, 37) = 14.03, *p* = .004 (corrected), η_p_^2^=.275, saccades between the second and the third syllables compared with words. The number of backward saccades elicited by nonwords and phantoms was not significantly different (*p* = .836). The number of forward saccades elicited by nonwords, however, was significantly higher than that elicited by phantoms (*p* = .021) and by words (*p* = .008).

**Table 2:**
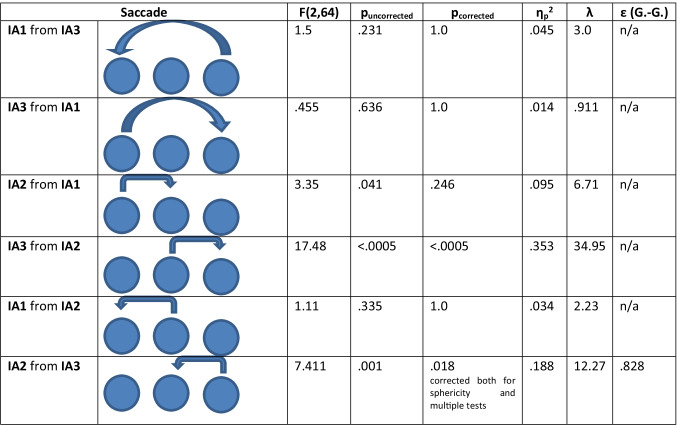
ANOVAs (repeated measures) on the number of saccades between different syllables in the triplets, with token *category* (words, phantoms, nonwords) as the main factor

### Discussion (Experiment 1)

The results show that overall recognition performance was substantially above chance: words were endorsed, while nonwords were rejected as legal constituents; phantoms tended to be accepted at above-chance levels, yet the likelihood of endorsing phantoms was lower than that of endorsing words. Importantly, an earlier study (Orbán et al., 2008) showed that in the visual modality, people fail to distinguish frequent pairs of symbols embedded in larger combinations of symbols from infrequent pairs that straddle boundaries of these combinations, once these combinations have been successfully extracted from a continuous visual input and committed to memory. Indeed, once constituents are learnt, knowledge of subelements of these constituents is also attenuated in linguistic material (Fiser & Aslin, 2005; Giroux & Rey, 2009). This phenomenon—referred to as the *embeddedness constraint—*might suggest that the phantoms in our experiment were recognized based on general adherence to learned statistical structure rather than on recognition of frequently co-occurring pairs, because the knowledge of pairs is supposedly attenuated by the end of the exposure. Consequently, it may be suggested that nonwords were rejected because they violated learnt statistical regularities, phantoms were endorsed based on statistical congruency with learnt regularities, while the higher acceptance rate for words than phantoms can be explained by the facilitatory effect of memory representations of whole triplets as words, presumably extracted and committed to memory as discrete constituents during the learning stage. This interpretation is in line with recent fMRI evidence (Ordin, Polyanskaya, & Soto, 2020a), where activation differences in the neural memory network were reported for presentation of both old and novel—but statistically congruent—triplets.

Although statistical learning is often considered to occur incidentally, cognitive processes typical of both implicit and explicit learning are activated as learning progresses (see discussion in Dienes et al., 1991; Perruchet & Pacton, 2006; Reber et al., 1991). As people learn regularities and constituents, they may become increasingly aware of them. If so, extracting those constituents from continuous sensory input that has already been presented multiple times and committed to memory is likely to be more conscious than extracting such constituents when they are first encountered at the initial stages of learning and still need to be memorized. Researchers have tested both explicit and implicit learning, and some studies have specifically examined the differences between tasks and underlying cognitive processes. As a rule, statistical learning has been shown to be more efficient when instructions are explicit (Kahta & Schiff, 2016; Laasonen et al., 2014; Reber et al., 1991; Schiff et al., 2017), but some researchers (Arciuli et al., 2014; Dienes et al., 1991) have reported that statistical learning performance does not vary with explicit versus implicit instructions. In this experiment, explicitly reporting feelings of confidence on each trial meant participants had to evaluate the likelihood of an error each time they decided whether the test token was a word. This suggests that they needed to consciously evaluate how aware they were of what they were assessing (what they had learnt). Larger differences between correct and incorrect responses reflect better ability to evaluate the likelihood of an error (Fleming & Lau, 2014; Pasquali et al., 2010; Schwiedrzik et al., 2011), and hence indicate the degree of conscious awareness of the learnt constituent. The data show that participants were more aware of having learnt words than phantoms, yet recognition of phantoms was still based on information that was subject to conscious processing to a higher degree than information needed to reject nonwords. Recognition of words was likely supported by memory representations of extracted triplets as holistic units from the sensory input. These units could be retrieved from memory as discrete constituents and consciously compared with statistically congruent tokens, raising the degree of conscious awareness when a presented token matched one of the memory representations. On trials where nonwords were presented as test tokens, a correct decision would have to be based on the *absence* of memory representations and/or violations of statistical structure. In this case, participants had to report their confidence in what they did not know or had not learnt, and it is not possible to have conscious awareness of a token or structure that has not been learnt. This could explain why the difference in confidence ratings assigned to correct versus incorrect trials with nonwords was the lowest difference found. Importantly, conscious awareness of what is being learnt is not required for successful statistical learning because, despite differences in the degree of conscious contributions to decisions, the proportion of correct responses on nonwords and words did not differ. At the same time, I cannot exclude the possibility that differences in the degree of conscious awareness in different types of recognition trials was due to the instructions given before familiarization rather than to differences in conscious awareness of what can be learnt. Participants were explicitly told to search for word-like units, and this could have tuned the relevant cognitive systems for detecting and memorizing holistic constituents at the expense of paying attention to structure (statistical regularities) or subconstituent units (frequently co-occurring syllabic pairs), raising conscious awareness of words during the familiarization phase. Both of these hypotheses could explain why the largest difference in confidence between correct and incorrect responses was found for word trials.

The eye-tracking data suggest that people check sequences of syllables in a pairwise fashion, with backward saccades made to verify that the two syllables are indeed parts of the same constituent. Saccades between boundary syllables were almost nonexistent, providing no evidence that boundaries were checked for consistency. In fact, overall, no evidence was observed for the use of boundary-finding mechanisms, at least at the recognition stage, where tokens were presented in isolation. Saccades between the second and the third syllables were more frequent for phantoms than words, signaling that participants found this transition more cognitively challenging and sought additional verification on phantom trials. By contrast, participants often made decisions on words without seeking additional verification. It seems that when activation of memory representations failed in the case of phantoms, participants felt obliged to verify that the penultimate–final syllables were indeed a frequently co-occurring pair. Again, it was not observed that people checked initial syllables on the backward saccades. It is possible that recognition of tokens presented in isolation and embedded into longer sequences of syllables may call for different cognitive mechanisms. Thus, a second experiment was set up to explore the cognitive mechanisms for online segmentation of holistic triplets, as opposed to the offline recognition of such units.

## Experiment 2

### Methods (Experiment 2)

Experiment 2 focused on extracting already learnt constituents embedded in longer sequences of seven syllables. Unlike Experiment 1, this paradigm encouraged participants to detect boundaries between discrete constituents in the sequence of syllables. In Experiment 1, all TPs between syllables within triplets were either equal to 0.5 (words and phantoms), or equal to 0 (nonwords) throughout all syllables presented on the screen. This could potentially discourage the use of bracketing mechanisms, which are based on detecting transitions that have relatively lower TPs than other transitions in a stream. In Experiment 2, whenever a word or a phantom was embedded in a longer sequence of seven syllables, the TP between the syllables straddling the word boundary was lower than the TP between syllables within phantoms and words. This created the necessary conditions to promote the use of a boundary-finding mechanism—that is, calculating *relative* TP differences between syllables, and aligning the word boundary with the TP that is lower than the neighboring intersyllabic transition.

#### Participants

Forty-three participants (age range: 18–35 years, *M* = 28, 31 females), who had not taken part in Experiment 1 but had the same profile, were recruited.

#### Material

The same material was used as in Experiment 1.

#### Procedure

The experiment consisted of two parts. The learning session was identical to that in Experiment 1. For the recognition test, seven syllables were presented on-screen along the central horizontal line, with 4 cm distance between syllables. Participants were told that the words from the novel language were embedded in some, but not all, of the seven-syllable sequences. They had to report whether the syllabic sequence contained a word from the language they had been exposed to, and then indicate (on a 6-point scale) how sure they were about their response.

**Fig. 6:**
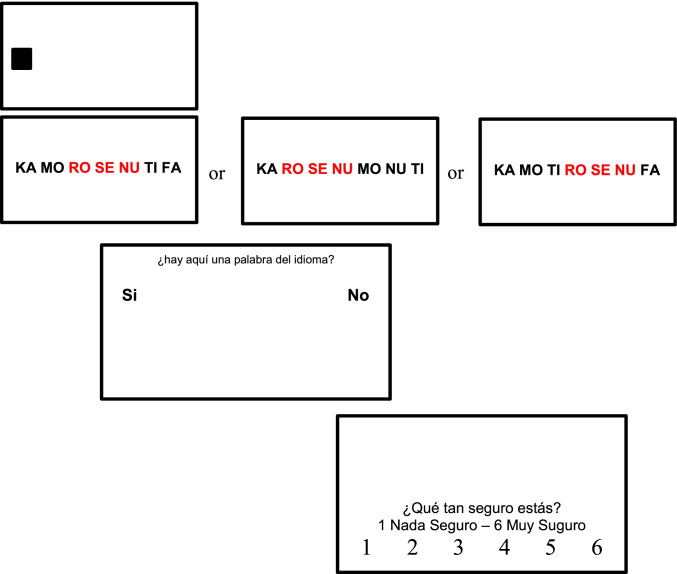
The schematic representation of a trial during the recognition test in Experiment 2. The questions—in Spanish—were (1) Is it a word from the language? ( “yes” or “no”), and (2) How sure are you? (scale from 1 = *not sure at all* to 6 = *very sure*). The embedded triplets were NOT highlighted in the actual experiment—they are highlighted here for the reader’s convenience

Each *word* was embedded in three test sequences, in the syllabic positions 2-3-4, 3-4-5, and 4-5-6. Each *phantom* and *nonword* was also embedded three times in the test sequences, again in three different positions within test sequences (108 trials in total). The order of trials was randomized for each participant. The eye-tracking machine was recalibrated before the recognition test, and the same 9-point grid calibration-validation procedure was repeated after each block of 36 trials. Each trial was initiated as soon as the participant fixated a black square marking where the first syllable of a seven-syllable sequence would appear. The sequence of syllables was displayed for 2,500 ms. There were no limits on response times. The structure of the trial and the experimental interface are presented in Fig. 6.

### Results (Experiment 2)

#### Accuracy and confidence analysis

**Fig. 7:**
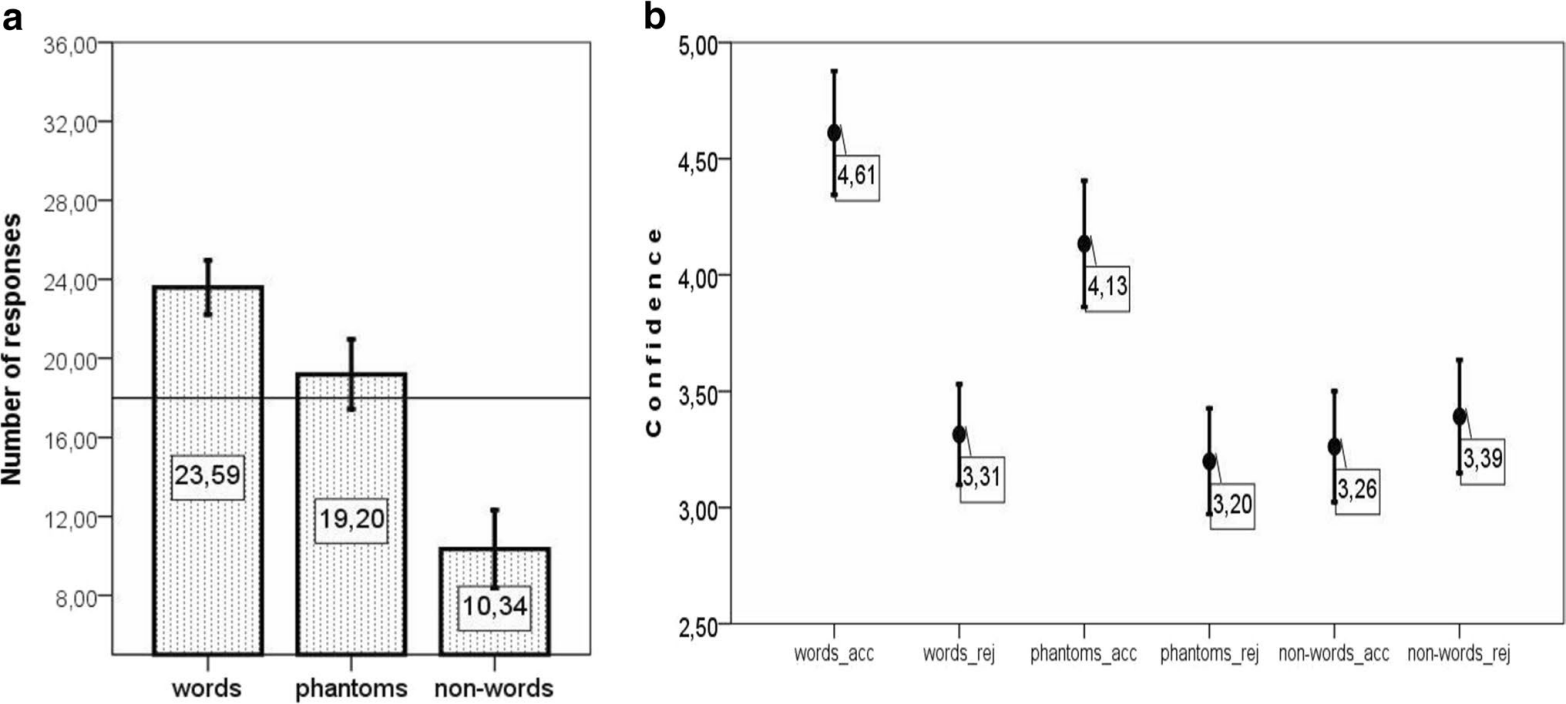
**a** Experiment 2: Accepted words, phantoms, and nonwords out of 36 trials per category of tokens. Error bars represent 95% CI. The horizontal line represents a chance level response. **b** Experiment 2: Averaged confidence on accepted (_acc) and rejected (_rej) words, phantoms, and nonwords. Error bars represent 95% CI. The scale is cropped. The range for confidence ratings is from 1 (*minimum confidence*) to 6 (*maximum confidence*)

Of 43 participants, two were excluded from further analyses for not following the task. A series of one-sample *t* tests showed that reporting whether the sequence contained a word, *t*(40) = 11.16, *p* < .0005, *M* = 7.59, [6.21, 8.96], or not, *t*(40) = −5.77, *p* < .0005, *M* = −5.66, [−7.64, −3.68], differed from what would be expected by chance. On trials with embedded phantoms, participants reported that the presented syllabic sequences did contain a word with a frequency significantly above that expected by chance, *t*(40) = 3.67, *p* = .001, *M* = 3.2, [1.44, 4.95]. As performance at the group level was different from what would be expected by chance, I suggest that the responses were not given randomly. The pattern is represented in Fig. 7a.

A repeated-measures ANOVA on the number of sequences, in which participants reported they detected embedded words, showed that whether the trial contained a word, a phantom or a nonword had a significant effect, *F*(2, 80) = 88.32, *p* < .0005, η_p_^2^ = .688 (λ = 176.64); Greenhouse–Geiser (ε = .855) was applied to correct for sphericity violations (corrected *p* values and uncorrected degrees of freedom are reported). Pairwise tests showed that the number of trials accepted differed significantly for embedded words and phantoms, *t*(40) = 5.28, *p* < .0005, *M* = 4.39, [2.71, 6.07], trials with and without embedded words, *t*(40) = 11.11, *p* < .0005, *M* = 13.24, [10.83, 15.65], and embedded phantoms and nonwords, *t*(40) = 8.96, *p* < .0005, *M* = 8.85, [6.86, 10.85]. The difference between correct endorsements on trials with words and correct rejections on trials with nonwords was insignificant, *t*(40) = −1.74, *p* = .18.

For the confidence analysis (see Fig. 7b), I first tested for significant differences in confidence assigned to accepted and rejected sequences (a) with embedded words, (b) with embedded phantoms, and (c) without words/phantoms. Two-tailed *t* tests showed that endorsed sequences received higher confidence than rejected sequences, if they contained words, *t*(40) = 12.58, *p* < .0005, *M* = 1.3, [1.01, 1.5], or phantoms, *t*(40) = 8.09, *p* < .0005, *M* = .93, [.7, 1.17]. No significant differences were observed between confidence ratings assigned to endorsed and rejected sequences that did not contain words or phantoms, *t*(40) = 1.0, *p* = .323, *M* = .13, [−.13, .34] (i.e., sequences in which nonwords were embedded).

The differences in confidence ratings assigned to accepted and rejected tokens per category were subject to a repeated-measures ANOVA with the Greenhouse–Geiser (ε = .781) for sphericity violations. This showed that the difference in confidence assigned to accepted and rejected tokens from different categories was significant, *F*(2, 80) = 76.32, *p* < .0005, η_p_^2^ = .279 (λ = 152.65), corrected p values and uncorrected degrees of freedom are reported. Further pairwise comparisons showed that the difference in confidence assigned to endorsed and rejected words was significantly larger than that for accepted and rejected phantoms, *t*(40) = 3.701, *p* = .002, *M* = .36, [.16, .56], as well as for accepted and rejected nonwords, *t*(40) = 9.63, *p* < .0005, *M* = 1.43, [1.13, 1.72). The difference in confidence assigned to accepted and rejected phantoms and to accepted and rejected nonwords was also significant, *t*(40) = 1.06, *p* < .0005, *M* = 1.06, [.86, 1.28].

#### Saccade analysis

**Fig. 8:**
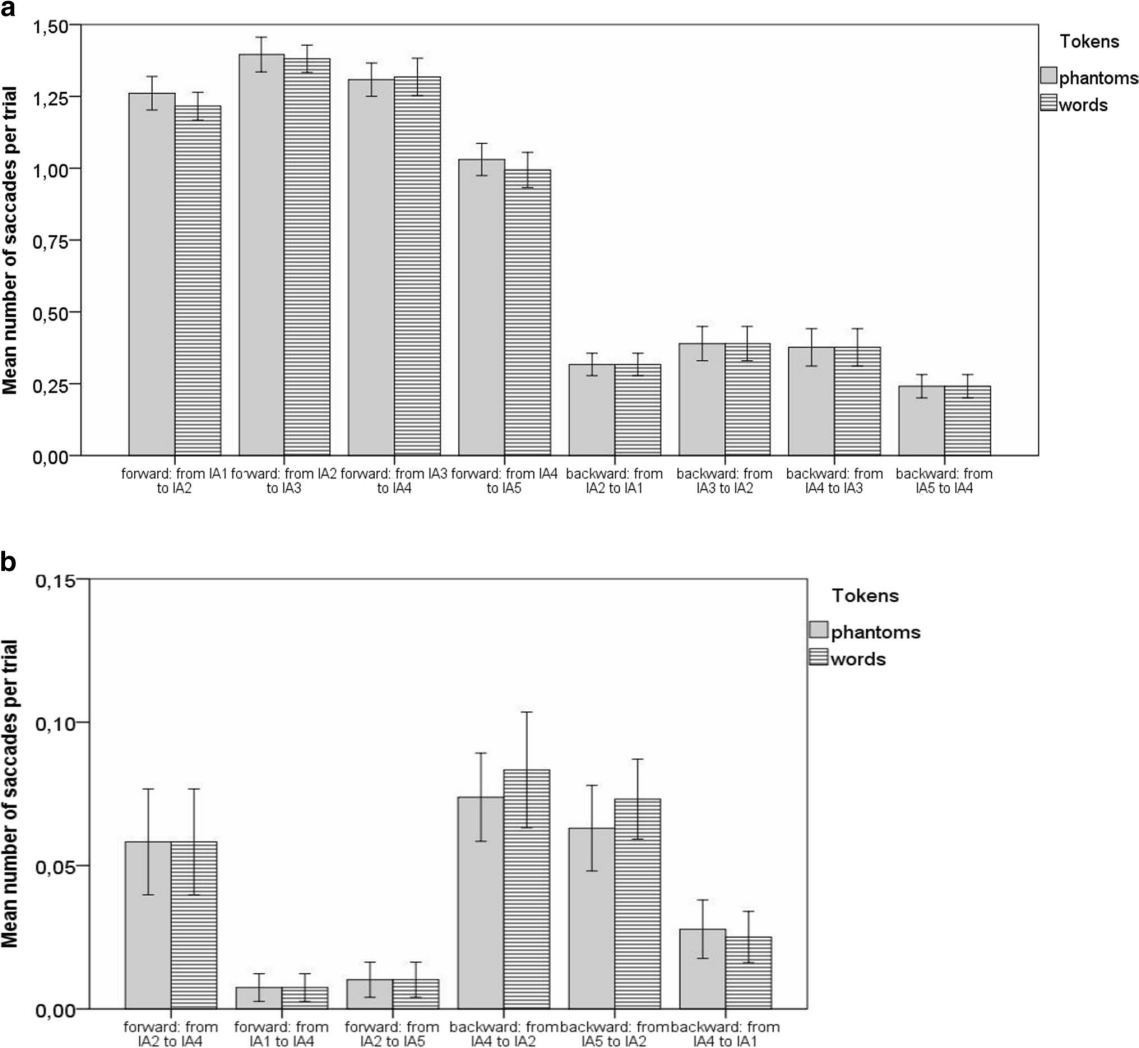
**a** Experiment 2: Mean number of saccades per trial between adjacent syllables (syllables 2-3-4 contain the words or phantoms). Error bars represent 95% CI. **b** Experiment 2: Mean number of saccades per trial between non-adjacent syllables (syllables 2-3-4 contain the words or phantoms). Error bars represent 95% CI

The protocol for data preprocessing was identical to that implemented in Experiment 1. To analyze the trials containing words and phantoms, syllable (and IA) positions were recoded. IA1 was the position preceding the initial syllable of the triplet, IAs 2, 3, and 4 contained correspondingly the first, second and third syllables of the triplets, while IA5 contained the syllable following the triplet. The mean numbers of saccades per trial between the recoded positions were calculated and plotted in Fig. 8a (pairwise saccades between adjacent syllables) and Fig. 8b (long-distance saccades between the item boundary syllables and beyond the boundary syllables). Forward pairwise saccades are more frequent than backward pairwise saccades. ANOVAs on each type of saccade with *category* (words and phantoms) as a factor did not reveal any differences in frequency of saccades for words and phantoms, suggesting a similar fixation sequence—mostly pairwise fixations—irrespective of the embedded token type. All tests yielded insignificant results (*p* > .4 before corrections for multiple tests and *p* = 1.0 after corrections). This pattern replicates that observed in Experiment 1, where participants saw the triplets in isolation. The figures show that long-distance saccades are very rare, while long-distance forward saccades are almost nonexistent. However, whenever backward saccades between nonadjacent syllables do occur, they tend to happen between syllables straddling the boundaries syllables (i.e., attracted to the low-TP transitions), providing some (albeit weak) support for some involvement of boundary-finding mechanisms. Short-distance saccades, however, were more frequent between syllables within triplets than between syllables straddling the triplet boundaries, suggesting that clustering mechanisms were dominant in extracting already learnt constituents from longer syllabic sequences, which is line with the interpretation of Experiment 1 data.

### Discussion (Experiment 2)

Accuracy results were similar to the pattern of results observed in Experiment 1. Sequences with embedded words were endorsed more frequently than sequences with embedded phantoms, although both sequences were endorsed significantly above chance. Sequences without embedded words and phantoms were rejected. No significant difference in accuracy was observed between rejected sequences without words and phantoms and accepted sequences with words.

This analysis of confidence ratings largely supports the interpretation of the data in Experiment 1. Conscious awareness of words as discrete constituents contributed to decisions on the word trials, responses on trials without embedded words or phantoms were made without conscious awareness. However, the degree of conscious contribution to decision-making did not modulate cognitive performance. This again suggests that statistical learning is possible without consciousness. Conscious awareness of words is grounded on explicit instructions given during the familiarization phase. Explicit instructions encouraged participants to pay attention to embedded sequences, making it more likely that people consciously processed these sequences. Conscious awareness was further strengthened by memory representations of these triplets when they were committed to memory in the course of familiarization. Unlike Experiment 1, Experiment 2 did not reveal any contribution from conscious awareness in recognition of phantoms. This likely resulted from differences between the two experiments. In Experiment 1, participants had to decide whether the syllable pairs within the phantoms were chunks. This decision could have been based either on the frequency of syllable co-occurrence or on the TPs between syllables within triplets. But since the triplets were presented in isolation, they did not provide information about relative differences between TPs that straddled triplet boundaries and those located within triplet boundaries. This meant that participants, in addition to frequency cues (the frequency with which two syllables had been encountered consecutively in the familiarization input), were forced to rely on some internal threshold to decide that TPs were overall high and should be located within triplet boundaries. By contrast, in Experiment 2, when the triplets were embedded into longer syllabic sequences, participants could rely on additional statistical cues—that is, relative differences in TPs (lower for syllabic pairs straddling the triplet boundaries and higher for within-triplet pairs). This increased information that can be extracted from TPs. Statistical regularities, which carried more information in Experiment 2 than in Experiment 1, were learnt without conscious awareness, and this resulted in a different pattern in confidence ratings on trials with phantoms in Experiments 1 and 2.

Analysis of the saccades showed that gaze transitions between adjacent syllables were more frequent than between nonadjacent syllables. Among all pairwise saccades, forward transitions were more frequent than backward transitions. No differences were observed in the saccades between syllables straddling triplet boundaries (i.e., low-probability transitions) and syllables within triplet boundaries (i.e., high-probability transitions). As low TPs did not attract a higher proportion of saccades than high TPs, the support for boundary-finding mechanisms (albeit weak) could only have been reflected in (relatively rare) long-distance saccades. However, whenever long-distance saccades were made, they tended to be backwards, going from the area marking the transition between the triplet-final and following syllables towards the transition between triplet-preceding and triplet-initial syllables. Although this can be viewed as evidence that the boundary-finding mechanism was at play, pairwise transitions were substantially more frequent, primarily implicating clustering segmentation mechanisms. It appears that boundary-finding mechanisms were employed rarely and only provided additional support. Absence of significant differences in saccades on trials with phantoms and words suggests that both types of tokens were extracted from a continuous speech based on the same cognitive segmentation mechanisms.

## General discussion

In Experiment 1, people had to evaluate the overall predictive strength between syllables, basing their decisions on high versus low TPs between the syllables presented on-screen or on whether adjacent syllables had frequently co-occurred in the familiarization input. In Experiment 2, people could additionally rely on the relative predictive strength of TPs between syllables. Regardless of whether people could rely on such relative relations (Experiment 2) or not (Experiment 1), words were recognized and endorsed at a higher rate than phantoms, while nonwords were rejected at the same rate as words were accepted. On trials in which words were presented, decision-making activated a higher degree of conscious awareness, yet conscious processing and awareness of test tokens is not a requirement for efficient statistical learning. As statistical learning can easily progress without conscious processing of information, building conscious representations of words appears to be a natural by-product in the evolution of representations: percepts of frequent syllable pairs become attenuated, and representations of triplets as holistic constituents are strengthened. This view of constructing conscious representations of words as a natural self-organization of percepts that emerge at earlier stages of exposure is in line with theoretical and computational models of statistical learning (Perruchet, 2005; Perruchet & Tillmann, 2010; Perruchet & Vinter, 1998, 2002; Poulin-Charronnat et al., 2016), which present statistical learning as extraction and accumulation of chunks based on associative learning principles and results in further merging of these chunks into larger constituents.

The saccade analysis clearly showed that the dominant cognitive mechanisms involved in statistical learning are based on clustering; only in Experiment 2 was there some weak evidence for engagement of boundary-finding mechanisms. This is surprising given that several earlier studies, including one of our own (e.g., Endress & Bonatti, 2007; Ordin, Polyanskaya, Soto, & Molinaro, 2020b), have shown that boundary-finding mechanisms underlie segmentation of continuous linguistic inputs. Importantly, most of these studies were performed in the auditory modality. Statistical learning is not a single mechanism, but rather an ability that is based on a set of mechanisms (Conway, 2020; Frost et al., 2015; Frost et al., 2019), and the combination of engaged mechanisms differ depending on sensory modality (Conway & Christiansen, 2006; Emberson et al., 2011). It is possible that in the auditory modality, detecting boundaries is more important than in the visual modality. Notably, speech segmentation in the auditory modality is modulated by pitch modifications (Ordin & Nespor, 2013, 2016; Shukla et al., 2007; Toro et al., 2009), lengthening of phrase-final syllables (Ordin et al., 2017), and inserting pauses between some constituents words (Buiatti et al., 2009; Endress & Mehler, 2009b). These cues facilitate boundary detection because they are often aligned with the initial or final syllables of constituents (the effect of prosodic cues that are not aligned with the boundaries of discrete constituents is either neutral or even impedes segmentation; Ordin & Nespor, 2013). Natural speech always contains prosodic cues, which facilitate detecting boundaries, especially when statistical and prosodic cues provide mutual support for boundary-finding mechanisms. By contrast, the current experiments were conducted in the visual modality, such that supplementary cues to constituent boundaries were not present in the familiarization input. This is likely to modulate the relative importance of clustering and boundary-finding mechanisms for segmentation purposes. The data suggest that clustering mechanisms prevail in segmentation of visual input.

It has been claimed by many researchers that statistical learning arises from a set of memory processes (McClelland et al., 1995; Perruchet & Vinter, 1998; Thiessen, 2017). Endress and Langus (2017), for example, argue that TPs are used to detect boundaries between constituents in continuous input, while memory is used to remember and recollect extracted constituents. This study suggests that memory mechanisms underlying segmentation might also differ depending on the perceptual modality engaged. Two classes of memory mechanisms were proposed: chaining memory (i.e., encoding which elements follow a given element within a sequence) and ordinal memory (i.e., encoding the position of smaller elements relative to the edges of a larger unit). Although humans use both memory mechanisms (see Endress & Wood, 2011; Henson, 1998, for a review; Marchetto & Bonatti, 2013; Ng & Maybery, 2002), the representation of linguistic sequences in the auditory modality has been shown to largely rely on positional mechanisms (Endress et al., 2005; Endress & Hauser, 2010). However, the results in the visual modality lead to the opposite conclusion. If the position of syllables within the triplets (the ordinal mechanism) mattered more than the chaining memory mechanism, no difference in accuracy would have been observed between words and phantoms. Compare, for example, the phantoms PASENU and ROSETI and the words ROSENU and PASETI. The syllable SE was always used in the intermediate position, while syllables RO and PA marked the triplet-initial, and syllables TI and NU marked the triplet-final position in both words and phantoms. Each syllable was used twice and always in the same ordinal position within statistical words and phantoms. If the participants had relied exclusively on an ordinal memory mechanism, the words and phantoms would have been encoded similarly, and the accuracy of responses on words and phantoms would not differ. Hence the tentative conclusion is that in the current experiment, sequencing of syllables within triplets was valued more than the position of syllables relative to the edges, which allowed words to be differentiated from phantoms. By contrast, in auditory material, ordinal memory mechanisms are probably evoked since the boundaries of the higher-order constituents are emphasized by prosodic cues that attract a listener’s attention to constituent edges.

To conclude, the data show that in the visual modality, segmentation of syllabic sequences relies mostly on clustering mechanisms. Differences between empirical results across studies are likely due to different underlying cognitive mechanisms and the differences in available cues in the auditory and visual modalities.

## Data Availability

Data for analysis and figures are available from the author upon request (leona.polyanskaya@gmail.com), and also deposited and publicly accessible on Figshare repository (10.6084/m9.figshare.17121326.v1).
